# The role of sleep and wakefulness in the recognition of emotional pictures

**DOI:** 10.1111/jsr.13695

**Published:** 2022-07-19

**Authors:** Giacomo Carollo, Giorgia Degasperi, Nicola Cellini

**Affiliations:** ^1^ Department of General Psychology University of Padova Padova Italy

**Keywords:** arousal, emotional memory, recognition test, rapid eye movement sleep, retention period, valence

## Abstract

Sleep has a beneficial effect on memory consolidation. However, its role in emotional memory is currently debated. Here, we investigate the role of sleep and a similar period of wakefulness on the recognition of emotional pictures and subjective emotional reactivity. Forty participants without any major physical, neurological or psychological condition were randomly assigned to the Sleep First Group or Wake First Group. The two groups underwent the encoding phase of an emotional images task with negative and neutral pictures at either 09:00 hours (Wake First Group) or 21:00 hours (Sleep First Group). Then participants performed an immediate recognition test (T1), and two delayed tests 12 hr (T2) and 24 hr (T3) later. Perceived arousal and valence levels were collected for each picture. Sleep parameters were recorded at participants' homes with a portable device. No differences were observed at T1, whereas at T2 the Sleep First Group showed a higher memory performance than the Wake First Group. At T3, performance decreased in the Sleep First Group (who spent the previous 12 hr awake), but not in the Wake First Group (who slept during the previous 12 hr). Overall, negative images were remembered better than neutral ones. We also observed a positive association between memory performance for negative items at the immediate test and the percentage of rapid eye movement sleep the night before the encoding. Our data confirm that negative information is remembered better over time than neutral information, and that sleep benefits the retention of declarative information. However, sleep seems not to preferentially improve emotional memory, although it may affect the encoding of negative information.

## 1 INTRODUCTION

Since the first mention of a “sleep effect”, which refers to the ability of sleep to enhance the recall of declarative memories (Jenkins & Dallenbach, 1924), a consistent pool of sleep research has been focused on its role in memory consolidation, leading to the development of several theoretical models (Born & Wilhelm, 2012; Genzel, Kroes, Dresler, & Battaglia, 2014; Tononi & Cirelli, 2006). For example, according to the Synaptic Downscaling Hypothesis (Tononi & Cirelli, 2006), slow‐wave sleep promotes the reduction of synaptic strength to optimize memory retention and facilitate successive learning. On the other hand, the Active System Consolidation Hypothesis (Born & Wilhelm, 2012) posits that information encoded during wakefulness undergoes a process of reactivation and reorganization during sleep, mainly involving hippocampal and neocortical networks.

However, for a long time, sleep and memory have been investigated without taking into account the possible contribution of emotional experience. Nevertheless, it has been demonstrated that emotions and emotion‐derived states (e.g. mood) can have a large impact on most brain and body functions, including memory formation and retention (Coughlin Della Selva, 2006; LaBar & Cabeza, 2006; McGaugh, 2004). More recently, given the consistent findings linking mood disorders with altered sleep patterns, researchers have begun to explore the relationship between sleep, emotional memory and affective regulation (for a comprehensive review on these topics, see Walker, 2009). Some evidence suggests that sleep preferentially enhances memory for emotional rather than neutral stimuli (Hu, Stylos‐Allan, & Walker, 2006; Wagner, Gais, & Born, 2001). In particular, negatively valenced information seems to be more resistant to forgetting, as it is often better remembered than positive or neutral material after a period of sleep deprivation (Tempesta et al., 2016; Walker, 2009). A discrete amount of research has been focused on the association between emotional memory and rapid eye movement sleep (REM). The focus on REM is justified by its unique physiology, and the evidence linking selective REM deprivation with an impaired encoding of emotional stimuli and altered emotional reactivity (Goldstein & Walker, 2014; Walker & van Der Helm, 2009). REM sleep is characterized by the activation of those cerebral regions implied in fear learning and broader forms of emotional processing during wakefulness, such as the amygdala, the anterior cingulate cortex, the entorhinal cortex, the ventromedial prefrontal cortex and the hippocampus (Tempesta, Socci, De Gennaro, & Ferrara, 2018). Its unique neurochemical environment is characterized by a reduced concentration of monoamines (e.g. adrenaline and noradrenaline) and by an increased concentration of acetylcholine. Although both neuromodulators are implied in synaptic plasticity and memory consolidation, the absence of monoamines during REM sleep is thought to allow the reactivation of emotional aspects of memories without enacting their associated physiological activation (Walker & van Der Helm, 2009). At the same time, cholinergic transmission is thought to play a critical role in high‐level cognitive activities such as learning and memory processes (Diekelmann & Born, 2010; Teber et al., 2004). Furthermore, the electroencephalogram (EEG) during REM sleep is dominated by the theta band, which is thought to be related to the consolidation of information acquired during the day (Hutchison & Rathore, 2015). Considering these peculiar characteristics, a well‐known theoretical model introduced by Walker & van del Helm (2009) proposes a possible role of REM sleep in supporting emotional regulation processes by strengthening salient memory representations while gradually reducing their affective tone over time.

Nonetheless, these hypothesized functions remain highly debated among scientists, who seem unable to find a clear agreement on the possible mechanisms linking sleep with memory consolidation and emotional processing (Lipinska, Stuart, Thomas, Baldwin, & Bolinger, 2019). Among the many questions that remain unanswered, it is still not clear how much of the “sleep effect” could be explained by the reduction of interference during sleep (i.e. the continuous flow of new sensory and cognitive information that is constantly processed by the brain during wakefulness; Jenkins & Dallenbach, 1924) or could be attributed to sleep‐exclusive active mechanisms supporting memory consolidation (Benson & Feinberg, 1977). Moreover, the role of REM sleep in emotional regulation and emotional memory consolidation has been confirmed in some studies (Hu et al., 2006; Van Der Helm et al., 2011; Wagner et al., 2001), but not in others (Baran, Pace‐Schott, Ericson, & Spencer, 2012; Cellini, Torre, Stegagno, & Sarlo, 2016; Groch, Wilhelm, Diekelmann, & Born, 2013). However, some of these results may be partially biased by the methodological differences across studies: for example, selective sleep deprivation and split‐night paradigms often produce incompatible results although being widely adopted to investigate the separate contributions of REM sleep and non‐REM sleep on memory consolidation (Wagner et al., 2001; Wagner, Fischer, & Born, 2002). Also, although deprivation studies offered a significant contribution to the effect of sleep on emotional processing (Tempesta et al., 2016), their observations are limited to the consequences of sleep loss (whether being partial or total) but do not allow to assess the direct contribution of specific sleep parameters on memory processes. Other researchers have obtained significant results through the use of nap paradigms (Cellini et al., 2016; Nishida & Walker, 2007), although this type of experimental design does not allow for examining the effect of whole nights of sleep on emotional memory consolidation and emotional reactivity. Moreover, while the effects of sleep on emotional memory consolidation have already been studied using a 1‐week longitudinal design, showing a negative association between sleep efficiency and negative picture discrimination (Cellini, Mercurio, & Sarlo, 2019), the monitoring of multiple consecutive nights with polysomnography (PSG)‐like accuracy has not yet been achieved for this aim. Lastly, although sleep has been extensively studied with regard to memory consolidation, far less interest has been directed to its effect on emotional information encoding, except for deprivation studies (Kaida, Niki, & Born, 2015).

Starting from this theoretical background, we conducted a study to assess the separate roles of sleep and wakefulness on emotional memory consolidation during 24 hr. Specifically, we used an emotional image recognition task and a portable sleep tracker to: (i) investigate the effect of nocturnal sleep compared with an equal period of daytime wakefulness on subjective emotional reactivity and memory performance; (ii) determine whether a positive effect of sleep on memory consolidation remains stable after a subsequent period of wakefulness; and (iii) explore the effect of nighttime sleep on the encoding of emotional information.

## 2 MATERIALS AND METHODS

### 2.1 Participants

Forty participants between the age of 18 and 34 years (17 M, 23 F; mean age ± standard deviation = 23.575 ± 3.161), and without any major physical, neurological or psychological condition took part in this study. Participants were randomly assigned to a Sleep First Group (SF; 8 M, 12 F) or a Wake First Group (WF; 9 M, 11 F), which provided for two different experimental conditions. All participants provided informed consent. The study protocol was approved by the local Ethics Committee.

### 2.2 Stimuli and task

Neutral and emotional images (*N* = 180) were selected from the EmoMadrid database (Carretié, Tapia, López‐Martín, & Albert, 2019), already validated for affective research, and further tested in a pilot sample of students from Padua University to confirm its validity in an Italian sample (*N* = 20; pilot data will not be presented in this manuscript). Each image in the database comes with a series of parameters, including Valence and Arousal values expressed on a 5‐point Likert scale ranging from −2 (very negative/very calm) to +2 (very positive/very arousing). The reason EmoMadrid has been chosen over the more popular International Affective Picture System (IAPS; Lang, Bradley, & Cuthbert, 2008) is that although the latter has been widely used for affective research, it presents some major issues regarding the obsolescence of some pictures and their cultural‐geographical background, which is mostly representative of USA (Carretié et al., 2019; Henrich, Heine, & Norenzayan, 2010).

The experimental task included an encoding phase and three memory tests (T1, T2, T3), all of which were built employing the PsychoPy software (Peirce et al., 2019). The Encoding set included 90 images (45 negatives + 45 neutral). During the encoding, each image was displayed on the screen for 2 s, after which participants were asked to report their perceived ratings of Valence and Arousal.

Memory tests included 60 images each, 30 of which (15 negatives + 15 neutral) had already been shown during the Encoding phase, while the other 30 (15 negatives + 15 neutral) were new. All test sets were built in order to balance thematic content (every set included the same number of images depicting animals, people, landscapes and objects) and Arousal/Valence levels (Negative Valence Mean ± Standard Deviation = −1.38 ± 0.08; Negative Arousal Mean ± Standard Deviation = 1.13 ± 0.04; Neutral Valence Mean ± Standard Deviation = 0.16 ± 0.06; Neutral Arousal Mean ± Standard Deviation = −0.03 ± 0.10). Every image appeared on the screen for 2 s, after which participants were asked to respond whether they had already seen it during the encoding or not, and to report their perceived ratings of valence and arousal. Memory performance was assessed through the d‐prime index. Specifically, we computed the Hit Rate (HR; the proportion of old pictures correctly identified as “already seen”) and False Alarm Rate (FAR; the proportion of new pictures mistakenly identified as “already seen”). From these two indices, according to Signal Detection Theory (Macmillan & Creelman, 2004), we calculated the discrimination index d‐prime as the difference between HR and FAR *z*‐scores, using the formula d‐prime = *z*HR − *z*FAR. For the d‐prime, HR and FAR, we also computed the change in memory performance between T2 and T1 as T2score/T1score*100, and the change in memory performance between T3 and T2 as T3score/T2score*100.

### 2.3 Sleep monitoring device

Sleep nights were monitored using the Dreem Headband (DH; Dreem SAS, Paris), a wearable device that has been validated as a portable alternative to PSG (Arnal et al., 2020). The headband comes with various sensors including five dry electrodes (O1, O2, FpZ, F7, F8) yielding seven bipolar EEG derivations (FpZ–O1, FpZ–O2, FpZ–F7, F8–F7, F7–01, F8–O2, FpZ–F8), a 3D accelerometer to measure the respiration rate and keep track of movements and positions, and a pulse oximeter to measure heart rate. The DH can collect and store physiological measures in real‐time during the night; raw recorded data are available from a dedicated cloud service. The DH uses a validated automatic sleep scoring to provide classical sleep metrics (e.g. sleep duration, time spent in different sleep stages).

### 2.4 Procedure

**FIGURE 1:**
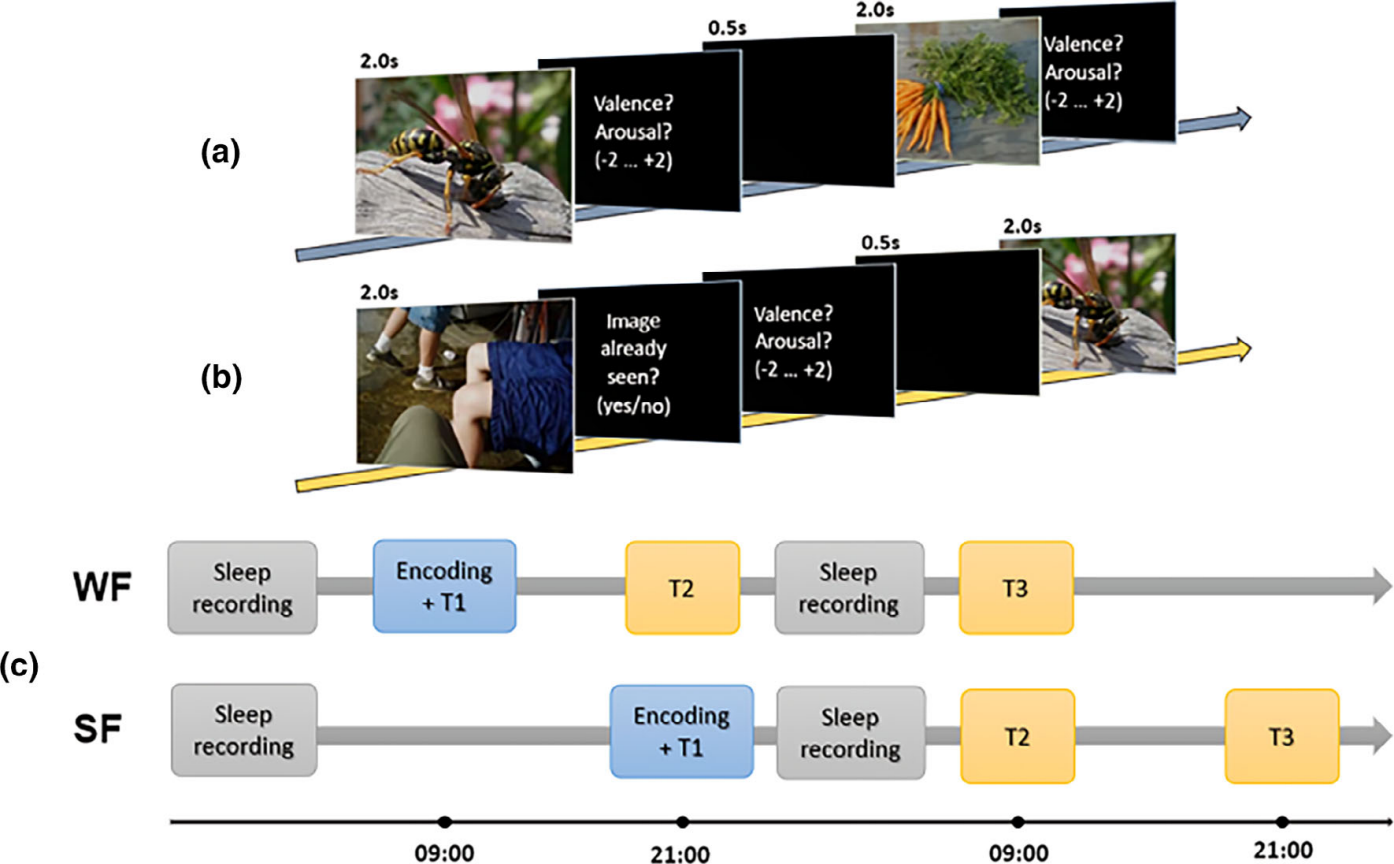
Experimental procedure of the (a) encoding phase and (b) tests. (c) Experimental protocol for the Wake First Group (WF) and the Sleep First Group (WF)

The whole procedure lasted 3 days for each participant (Figure 1), with a different distribution of tasks for the SF and WF. Each participant was given detailed oral and written instructions on how to record sleep nights and complete experimental tasks.

On the first day, all participants were asked to complete a series of online questionnaires in order to obtain basic demographics (age, gender, occupation), check for the presence of anxiety or depressive symptoms with the Hospital Anxiety and Depression Scale (HADS; Zigmond & Snaith, 1983), investigate global sleep quality through the Pittsburgh Sleep Quality Index (PSQI; Buysse, Reynolds III, Monk, Berman, & Kupfer, 1989), and circadian preferences through the ultra‐short version of the Munich ChronoType Questionnaire (MCTQ; Ghotbi et al., 2020) and the Morningness–Eveningness Questionnaire reduced version (MEQ‐r; Natale, Esposito, Martoni, & Fabbri, 2006), and to record the first night of sleep.

Beginning from the second day, instructions changed depending on the experimental condition: for the WF, initial learning (Encoding) took place at 09:00 ± 1:00 hours, followed by an immediate memory test (T1), while the second test (T2) had to be completed at 21:00 ± 1:00 hours. After that, participants had to record the second night of sleep and complete the last memory test (T3) at 09:00 ± 1:00 hours on the following day. Instructions for the SF were similar, except for the fact that experimental tasks were shifted by a 12‐hr interval so that initial learning would take place just before going to sleep. Thus, the Encoding phase and T1 had to be completed at 21:00 ± 1:00 hours on the second day, while the remaining tests (T2 and T3) were scheduled, respectively, for 09:00 ± 1:00 hours and 21:00 ± 1:00 hours on the following day, after the second night of sleep was recorded. To prevent any order effects, all tests were counterbalanced between participants for a total of six possible combinations. Moreover, the presentation order of pictures was randomized for each task. Before each experimental session, participants had to complete a short series of questionnaires that changed depending on the time of the day. The morning questionnaires included a PSQI (Buysse et al., 1989) to test sleep quality related to the previous night, a Samn–Perelli Scale (Samn & Perelli, 1982) and a Stanford Sleepiness Scale (Hoddes, Zarcone, Smythe, Phillips, & Dement, 1973) to, respectively, investigate vigilance and tiredness levels, whereas the evening questionnaires included only a Samn–Perelli and a Stanford Sleepiness Scale.

### 2.5 Statistical analysis

Demographics (age, gender), psychological and sleep parameters of the two groups were compared using independent *t*‐tests and *χ* ^2^‐tests. For each comparison, we reported Cohen's *d* as a measure of effect size.

Changes in memory performances across testing sessions have been analysed using the following plan. First, for each variable of interest we conducted an omnibus mixed‐ANOVA with testing Session (T1, T2, T3) and type of image (Negative, Neutral) as within‐subjects factors, and Group (SF, WF) as between‐subject factor.

Then, to test the first experimental hypothesis (i.e. lower forgetting after a night of sleep), we conducted a mixed‐ANOVA using changes scores from T1 to T2 as dependent variables, with Type of image (Negative, Neutral) as within‐subjects factor and Group (SF, WF) as between‐subjects factor. To test our second experimental hypothesis (i.e. the memory performance after a night of sleep remains stable after subsequent wake), we conducted another mixed‐ANOVA using changes scores from T2 to T3 as dependent variables, Type of image (Negative, Neutral) as within‐subjects factor and Group (SF, WF) as between‐subjects factor.

To assess the changes in emotional reactivity (Arousal and Valence), we conducted two separate omnibus mixed‐ANOVA with testing Session (Encoding, T1, T2, T3) and Type of image (Negative, Neutral) as within‐subjects factors, and Group (SF, WF) as between‐subjects factor. For all the ANOVAs, we reported *η* ^2^ *p* as a measure of effect size for the main factors and interactions, and we reported both uncorrected and Holm test post hoc analysis, using Cohen's *d* as a measure of effect size for post hoc comparisons.

The relationship between sleep parameters and performance as well as Arousal and Valence has been explored separately for the two groups using Pearson's correlations. For WF, we explored the relationship between sleep parameters recorded the night before encoding and the variables of interest, such as d‐prime, valence and arousal ratings at T1. For the SF, we explored the relationship between sleep parameters recorded the night after encoding and the change in performance from T1 to T2. A value of *p* < 0.05 was used as the significant level.

Besides null‐hypothesis significance testing, we also employed Bayesian statistics to estimate the probability of the alternative hypothesis being true given the data. Specifically, we reported the Bayes Factor (BF_10_), with values larger than three indicating moderate evidence for the alternative hypothesis (H1), and BF_10_ values lower than 0.3 moderately supporting the null hypothesis (H0; Jarosz & Wiley, 2014). For the ANOVAs, BF_10_ was computed using the across‐matched models approach. All the analyses were conducted using JASP version 0.16.2 (JASP Team, 2022). The study was not preregistered.

## 3 RESULTS

### 3.1 Demographic variables

No significant difference was found in any demographic or psychological variables between the two groups (Table 1).

**TABLE 1:** Means ± standard deviations of demographic and psychological variables in the two groups

	SF (*n* = 20)	WF (*n* = 20)	*t*	*p*	Cohen's *d*	BF_10_
Age (years)	24.07 ± 3.04	23.73 ± 3.19	0.348	0.730	0.110	0.325
Gender (F/M)	12/8	11/9	0.102*	0.749	0.205^a^	0.390
PSQI	5.20 ± 2.19	5.65 ± 2.01	−0.677	0.502	−0.214	0.371
MSFsc	4.36 ± 0.54	4.30 ± 0.45	0.344	0.733	0.079	0.317
MEQ‐r	12.25 ± 3.39	14.00 ± 4.00	−1.493	0.144	−0.472	0.741
HADS‐D	4.90 ± 2.49	5.00 ± 3.16	−0.111	0.912	−0.035	0.310
HADS‐A	7.70 ± 3.87	7.45 ± 3.85	0.205	0.839	0.065	0.314

BF, Bayesian factor; HADS‐A, Hospital Anxiety and Depression Scale‐anxiety; HADS‐D, Hospital Anxiety and Depression Scale‐depression; MEQ‐r, Morningness–Eveningness Questionnaire reduced version; MSFsc, timing of mid‐sleep on free days corrected for sleep‐debt; PSQI, Pittsburg Sleep Quality Index; SF, Sleep First Group; WF, Wake First Group.

^a^ Log odds ratio.

* *χ* ^2^‐value.

### 3.2 Valence and arousal ratings

**FIGURE 2:**
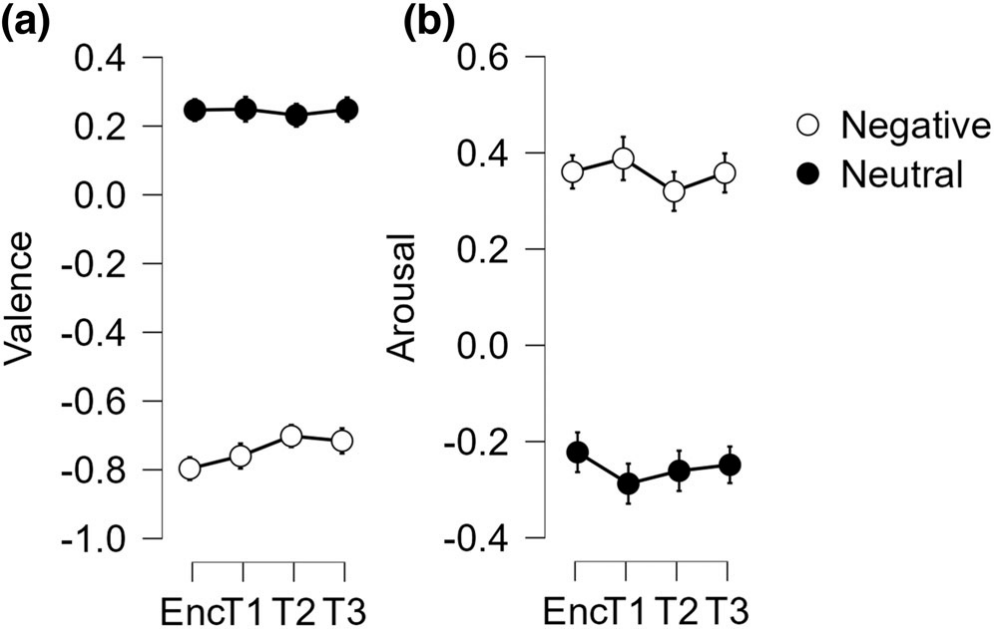
(a) Valence and (b) arousal ratings as a function of the type of stimuli (negative and neutral pictures) at the encoding (Enc) and the three testing sessions (T1, T2, T3). Error bars represent the standard error of the mean

The analysis of the Valence ratings (Figure 2a) showed only a significant Type main effect (*F*_1,38_ = 298.34, *p* < 0.001, *η* ^2^ *p* = 0.89; BF_10_ = ∞), with higher Valence ratings for neutral than negative pictures. We also observed a significant Type × Session interaction (*F*_1,38_ = 4.01, *p* = 0.009, *η* ^2^ *p* = 0.096; BF_10_ = 0.014), with Valence for Negative items increasing from the Encoding to T2 (*p*_holm_ = 0.003; Cohen's *d* = −0.347; BF_10_ = 108.96) and trending for T3 (*p*_holm_ = 0.065; Cohen's *d* = −0.295; BF_10_ = 6.078), but not from Encoding to T1 (*p*_holm_ = 0.912; Cohen's *d* = −0.134; BF_10_ = 0.631), and from T1 to T2 and T3 (both *p*_holm_ > 0.372; Cohen's *d* < −0.161; BF_10_ < 1.382). No change was observed in Neutral stimuli (all *p* > 0.999). All the other effects and interactions were not significant (all *p* > 0.084).

Similar results were observed for the Arousal ratings (Figure 2b), with a significant Type main effect (*F*_1,38_ = 104.70, *p* < 0.001, *η* ^2^ *p* = 0.734; BF_10_ = 2.002e^15^), with higher arousal ratings for negative than neutral pictures. All the other effects and interactions were not significant (all *p* > 0.130).

### 3.3 D‐prime

**FIGURE 3:**
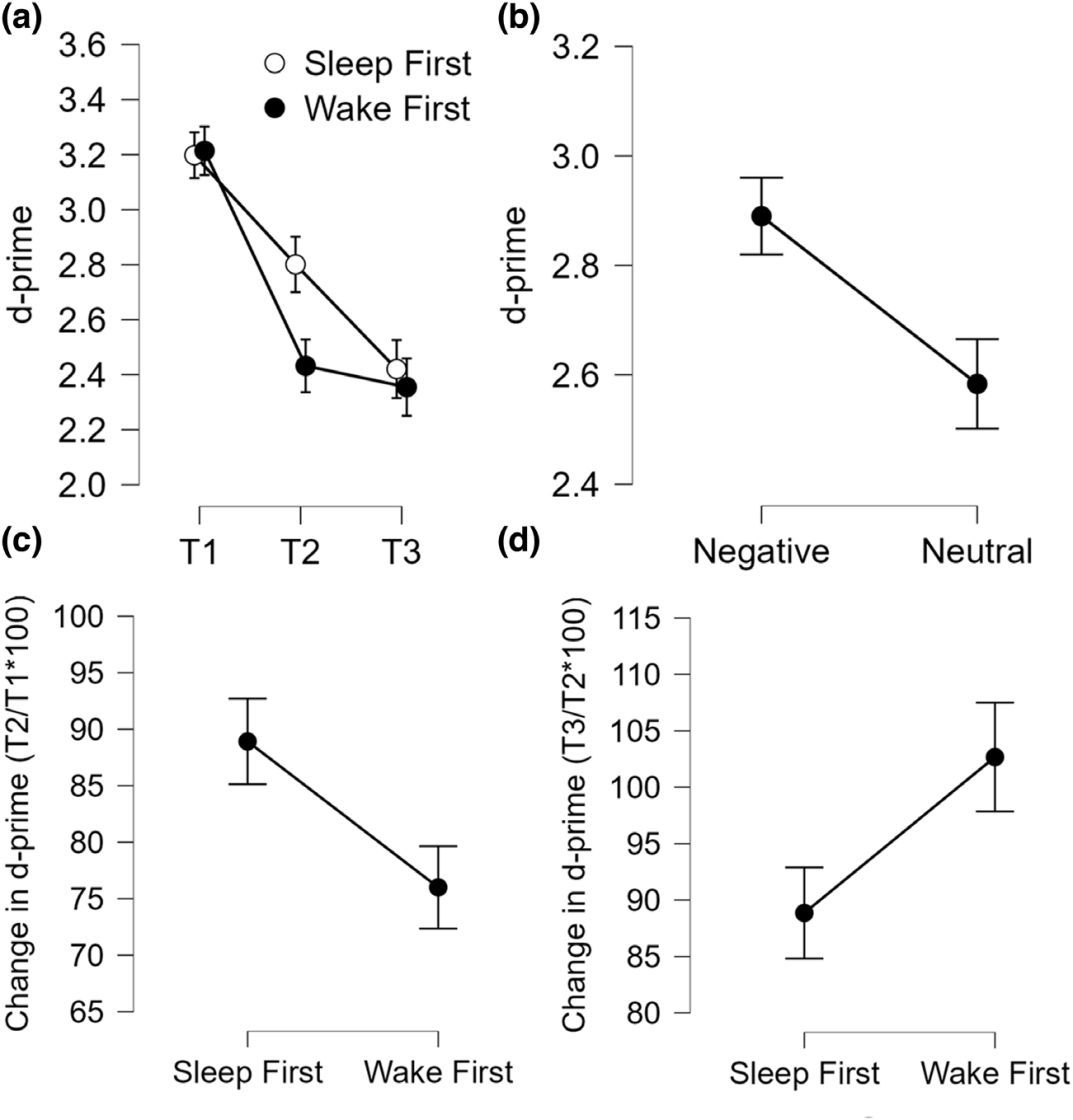
d‐prime. (a) d‐prime scores across the three testing sessions in the two groups; (b) overall d‐prime scores for negative and neutral pictures; (c) change in d‐prime scores from T1 to T2 (as T2/T1*100) in the two groups; (d) change in d‐prime scores from T2 to T3 (as T3/T2*100) in the two groups. Error bars represent standard errors of the mean

Results of the omnibus ANOVA examining the d‐prime across sessions in the two showed a significant Type effect (*F*_1,38_ = 19.13, *p* < 0.001, *η* ^2^ *p* = 0.34; BF_10_ = 170.026; Figure 3b), with better memory performance for Negative items. We also observed an expected Session effect (*F*_1,76_ = 47.31, *p* < 0.001, *η* ^2^ *p* = 0.56; BF_10_ = 9.621e^15^), with a linear decrease in d‐prime from T1 to T2 (*p*_holm_ < 0.001; Cohen's *d* = 0.939; BF_10_ = 6.345e^6^) and from T2 to T3 (*p*_holm_ = 0.006; Cohen's *d* = 0.365; BF_10_ = 9.772). Although the Group effect was not significant (*F*_1,38_ = 1.77, *p* = 0.285, *η* ^2^ *p* = 0.03; BF_10_ = 0.405), we observed a trend for the critical Group × Session interaction (*F*_1,76_ = 2.726, *p* = 0.072, *η* ^2^ *p* = 0.07; BF_10_ = 1.008; Figure 3a). Exploratory post hoc comparison of this interaction showed no differences at T1 (*p*_uncorrected_ = 0.882, *p*_holm_ > 0.999; Cohen's *d* = −0.025; BF_10_ = 0.312) and T3 (*p*_uncorrected_ = 0.701, *p*_holm_ > 0.999; Cohen's *d* = 0.105; BF_10_ = 0.328), and a higher d‐prime in the SF at T2 (*p*_uncorrected_ = 0.024; Cohen's *d* = 0.587; BF_10_ = 1.177). The latter result became non‐significant when corrected for multiple‐comparisons (16 comparisons, *p*_holm_ = 0.119). All the other main comparisons or interactions were not significant (all *p* > 0.487).

The ANOVA on change in d‐prime from T1 to T2 showed a significant Group effect (*F*_1,38_ = 4.46, *p* = 0.041, *η* ^2^ *p* = 0.11; BF_10_ = 1.666; Figure 3c), with higher d‐prime for the SF. Both the Type effect (*F*_1,38_ = 2.40, *p* = 0.130, *η* ^2^ *p* = 0.06; BF_10_ = 0.657) and the interaction Type × Group (*F*_1,38_ = 1.09, *p* = 0.302, *η* ^2^ *p* = 0.03; BF_10_ = 0.463) were not significant.

The ANOVA on change in d‐prime from T2 to T3 showed again a significant Group effect (*F*_1,38_ = 4.33, *p* = 0.044, *η* ^2^ *p* = 0.10; BF_10_ = 1.249; Figure 3d), but this time the change was higher for the WF. Again, both the Type effect (*F*_1,38_ = 0.14, *p* = 0.715, *η* ^2^ *p* < 0.01; BF_10_ = 0.246) and the interaction Type × Group (*F*_1,38_ = 0.20, *p* = 0.662, *η* ^2^ *p* = 0.01; BF_10_ = 0.323) were not significant.

### 3.4 Hit Rate

**FIGURE 4:**
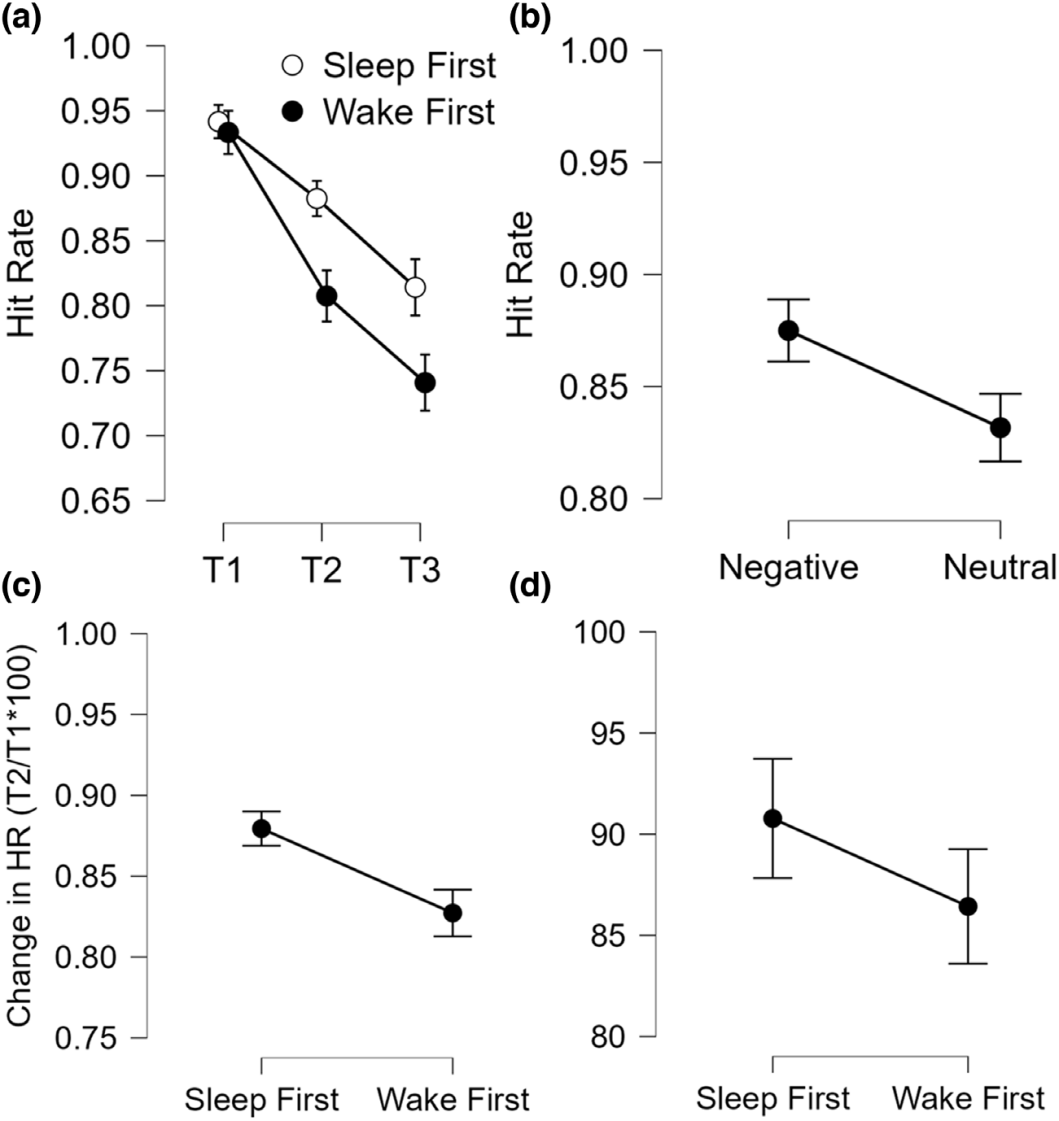
Hit Rate (HR). (a) HR scores across the three testing sessions in the two groups; (b) overall HR scores for negative and neutral pictures; (c) change in HR scores from T1 to T2 (as T2/T1*100) in the two groups; (d) change in HR scores from T2 to T3 (as T3/T2*100) in the two groups. Error bars represent standard errors of the mean

The omnibus ANOVA on HR showed a significant Type effect (*F*_1,38_ = 10.79, *p* = 0.002, *η* ^2^ *p* = 0.22; BF_10_ = 26.743; Figure 4b), with better memory recognition for Negative items. We also observed a Session effect (*F*_1,76_ = 41.89, *p* < 0.001, *η* ^2^ *p* = 0.52; BF_10_ = 8.043e^15^), with a linear decrease in d‐prime from T1 to T2 and from T2 to T3 (both *p*_holm_ < 0.001; Cohen's *d* > 0.556; BF_10_ > 422.439). We observed a main Group effect (*F*_1,38_ = 4.23, *p* = 0.047, *η* ^2^ *p* = 0.10; BF_10_ = 1.391), but the critical Group × Session interaction was not significant (*F*_1,76_ = 2.35, *p* = 0.103, *η* ^2^ *p* = 0.06; BF_10_ = 0.948; Figure 4a). Exploratory post hoc comparison of this interaction showed no differences at T1 (*p*_uncorrected_ = 0.498, *p*_holm_ = 0.996; Cohen's *d* = 0.069; BF_10_ = 0.372) and T3 (*p*_uncorrected_ = 0.091, *p*_holm_ = 0.274; Cohen's *d* = 0.604; BF_10_ = 0.995), and a higher HR in the SF at T2 (*p*_uncorrected_ = 0.039; Cohen's *d* = 0.617; BF_10_ = 1.810). The latter result became non‐significant when corrected for multiple‐comparisons (*p*_holm_ = 0.194). All the other main comparisons or interactions were not significant (all *p* > 0.318).

The ANOVA on change in HR from T1 to T2 showed a significant Group effect (*F*_1,38_ = 4.31, *p* = 0.045, *η* ^2^ *p* = 0.10; BF_10_ = 1.735; Figure 4c), with higher HR for the SF. Both the Type effect (*F*_1,38_ = 0.929, *p* = 0.341, *η* ^2^ *p* = 0.02; BF_10_ = 0.331) and the interaction Type × Group (*F*_1,38_ = 0.03, *p* = 0.875, *η* ^2^ *p* < 0.01; BF_10_ = 0.303) were not significant.

The ANOVA on change in HR from T2 to T3 showed no significant Group (*F*_1,38_ = 0.01, *p* = 0.915, *η* ^2^ *p* < 0.01; BF_10_ = 0.421; Figure 3d), Type (*F*_1,38_ = 0.11, *p* = 0.738, *η* ^2^ *p* < 0.01; BF_10_ = 1.594) or Type × Group (*F*_1,38_ = 0.31, *p* = 0.580, *η* ^2^ *p* < 0.01; BF_10_ = 0.374) effect.

### 3.5 False Alarm Rate

**FIGURE 5:**
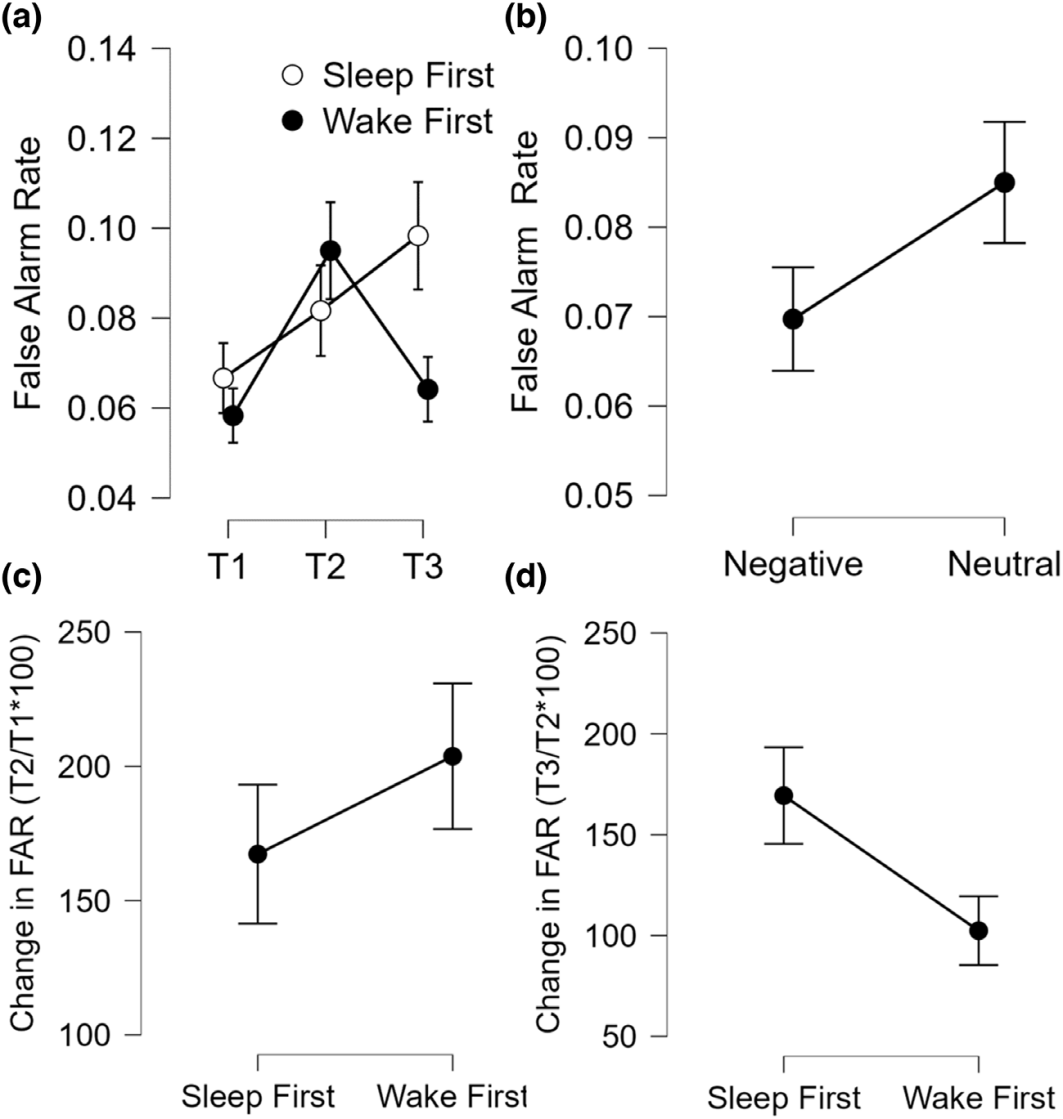
False Alarm Rate (FAR). (a) FAR scores across the three testing sessions in the two groups; (b) overall FAR scores for negative and neutral pictures; (c) change in FAR scores from T1 to T2 (as T2/T1*100) in the two groups; (d) change in FAR scores from T2 to T3 (as T3/T2*100) in the two groups. Error bars represent standard errors of the mean

The omnibus ANOVA on FAR showed a significant Type effect (*F*_1,38_ = 8.06, *p* = 0.007, *η* ^2^ *p* = 0.18; BF_10_ = 1.892; Figure 5b), with higher FAR for Neutral items. We also observed a Session effect (*F*_1,76_ = 4.61, *p* = 0.013, *η* ^2^ *p* = 0.11; BF_10_ = 5.840), with an increase in HR from T1 to T2 (*p*_uncorrected_ = 0.004, *p*_holm_ = 0.013; Cohen's *d* = −0.585; BF_10_ = 11.750) and from T1 to T3 (*p*_uncorrected_ = 0.021, *p*_holm_ = 0.041; Cohen's *d* = −0.352; BF_10_ = 1.320), but not from T2 to T3 (*p*_uncorrected_ = 0.541, *p*_holm_ = 0.541; Cohen's *d* = 0.133; BF_10_ = 0.165). Although we did not observe a main Group effect (*F*_1,38_ = 1.27, *p* = 0.267, *η* ^2^ *p* = 0.03; BF_10_ = 0.374), the Group × Session interaction was significant (*F*_1,76_ = 4.36, *p* = 0.016, *η* ^2^ *p* = 0.10; BF_10_ = 2.890; Figure 5a). Post hoc comparisons showed no differences at T1 (*p*_uncorrected_ = 0.369, *p*_holm_ > 0.999; Cohen's *d* = 0.156; BF_10_ = 0.429) and T2 (*p*_uncorrected_ = 0.263, *p*_holm_ > 0.999; Cohen's *d* = −0.250; BF_10_ = 0.441), but a higher FAR in the SF at T3 (*p*_uncorrected_ = 0.019; Cohen's *d* = 0.642; BF_10_ = 3.043). The latter result became non‐significant when corrected for multiple‐comparisons (*p*_holm_ = 0.178). All the other main comparisons or interactions were not significant (all *p* > 0.318).

The ANOVA on change in FAR from T1 to T2 showed a significant Type effect (*F*_1,38_ = 4.77, *p* = 0.035, *η* ^2^ *p* = 0.11; BF_10_ = 1.862; Figure 5c), with higher change for the Neutral picture. Both the Group effect (*F*_1,38_ = 0.757, *p* = 0.390, *η* ^2^ *p* = 0.02; BF_10_ = 0.395) and the interaction Type × Group (*F*_1,38_ = 1.68, *p* = 0.203, *η* ^2^ *p* = 0.04; BF_10_ = 0.634) were not significant.

The ANOVA on change in FAR from T2 to T3 showed a trend for a Group effect (*F*_1,38_ = 3.94, *p* = 0.054, *η* ^2^ *p* = 0.09; BF_10_ = 1.451; Figure 5d), with greater change for the SF, but no significant Type effect (*F*_1,38_ = 0.281, *p* = 0.599, *η* ^2^ *p* = 0.01; BF_10_ = 0.237) and Type × Group effect (*F*_1,38_ = 0.311, *p* = 0.580, *η* ^2^ *p* = 0.01; BF_10_ = 0.306).

### 3.6 Sleep patterns during the experimental night

The two groups did not show any difference in their sleep pattern during the experimental night (between T1 and T2 for the SF, and between T2 and T3 for the WF; Table 2). Exploring a potential association between sleep parameters during the experimental night and change in performance in SF revealed no significant associations.

**TABLE 2:** Means ± standard deviations of sleep variables during the experimental night

	SF (*n* = 20)	WF (*n* = 20)	*t*	*p*	Cohen's *d*	BF_10_
Time in bed (min)	421.31 ± 52.46	430.91 ± 60.14	−0.53	0.594	−0.17	0.347
Total sleep time (min)	392.73 ± 50.20	403.53 ± 57.32	−0.63	0.530	−0.20	0.362
Sleep‐onset latency (min)	14.09 ± 16.39	11.65 ± 9.22	0.58	0.565	0.18	0.353
Wake after sleep onset (min)	4.70 ± 5.14	7.50 ± 16.12	−0.74	0.464	−0.23	0.384
Sleep efficiency (%)	95.49 ± 0.05	95.40 ± 0.04	0.06	0.955	0.07	0.314
N1 (min)	1.47 ± 1.74	0.72 ± 1.09	1.63	0.110	0.52	0.879
N2 (min)	174.42 ± 46.57	181.80 ± 46.42	−0.50	0.619	−0.16	0.341
N3 (min)	108.58 ± 31.70	117.55 ± 24.41	−1.00	0.322	−0.32	0.460
REM (min)	114.20 ± 23.25	107.90 ± 33.30	0.69	0.492	0.22	0.374
N1 (%)	0.41 ± 0.62	0.18 ± 0.27	1.50	0.141	0.48	0.750
N2 (%)	44.50 ± 10.09	44.87 ± 8.30	−0.13	0.896	−0.04	0.311
N3 (%)	27.49 ± 7.55	29.29 ± 5.17	−0.88	0.385	−0.28	0.420
REM (%)	29.42 ± 6.90	26.77 ± 7.31	1.18	0.246	0.37	0.535

BF, Bayesian factor; REM, rapid eye movement sleep; SF, Sleep First Group; WF, Wake First Group.

### 3.7 Correlations between the sleep in the night before the encoding and performance at the encoding in the WF

The two groups did not show any difference in their sleep pattern during the night before the experimental day (Table 3).

**TABLE 3:** Means ± standard deviations of sleep variables during the night before the encoding

	SF (*n* = 20)	WF (*n* = 20)	*t*	*p*	Cohen's *d*	BF_10_
Time in bed (min)	426.45 ± 83.81	442.71 ± 53.60	−0.73	0.469	−0.23	0.382
Total sleep time (min)	397.73 ± 81.44	413.45 ± 55.34	−0.71	0.479	−0.23	0.378
Sleep‐onset latency (min)	12.51 ± 8.30	14.73 ± 7.12	−0.91	0.368	−0.29	0.429
Wake after sleep onset (min)	8.28 ± 9.66	5.75 ± 5.58	1.03	0.318	0.32	0.464
Sleep efficiency (%)	94.4 ± 3.9	94.7 ± 3.9	−0.35	0.731	−0.11	0.324
N1 (min)	1.6 ± 3.14	1.43 ± 1.81	2.16	0.830	0.07	0.315
N2 (min)	194.8 ± 49.6	189.58 ± 41.16	0.36	0.719	0.12	0.325
N3 (min)	94.98 ± 31.18	116.2 ± 36.10	−1.99	0.054	−0.63	1.433
REM (min)	111.23 ± 33.20	111.45 ± 31.69	−0.02	0.983	−0.01	0.309
N1 (%)	0.36 ± 0.66	0.35 ± 0.43	0.08	0.938	0.03	0.310
N2 (%)	49.61 ± 9.79	45.93 ± 8.50	1.27	0.212	0.40	0.584
N3 (%)	23.38 ± 8.07	28.22 ± 7.79	−1.93	0.061	−0.61	1.313
REM (%)	27.99 ± 5.41	26.81 ± 5.96	0.66	0.515	0.37	0.367

BF, Bayesian factor; REM, rapid eye movement sleep; SF, Sleep First Group; WF, Wake First Group.

**FIGURE 6:**
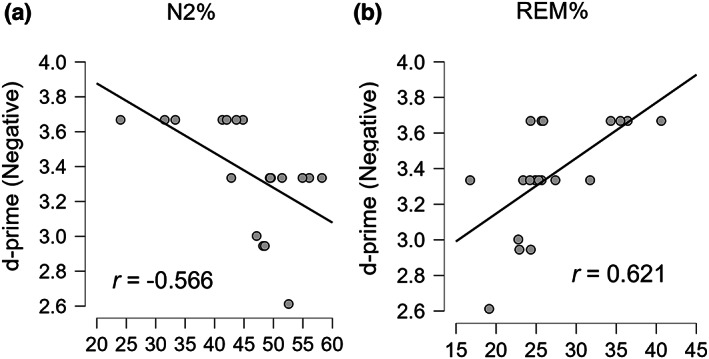
Correlations between previous night and encoding level. (a) Negative correlation between previous night N2% and d‐prime for negative items. (b) Positive correlation between previous night rapid eye movement (REM)% and d‐prime for negative items

Exploring the association between sleep parameter the night before the encoding and performance at the encoding only in the WF, only for the negative items, we observed a negative association between d‐prime and N2% (*r* = −0.566, *p* = 0.009; BF_10_ = 6.523; Figure 6a) and a positive one with REM% (*r* = 0.621, *p* = 0.003; BF_10_ = 14.876; Figure 6b). These associations were not present for neutral items.

### 3.8 Adaptation night effect

To control for a potential adaptation night effect (i.e. sleeping with a wearable device), we conducted several mixed‐models ANOVAs with the sleep parameters of interest as the dependent variable (Tables 2 and 3), with Night (first, second) as within‐subjects factor and Group as between‐subjects factor. The only significant comparison was a Night effect for N3% (*F*_1,38_ = 4.98, *p* = 0.032, *η* ^2^ *p* = 0.12; BF_10_ = 1.744), with participants spending more time asleep in N3 during the second night, but Group (*F*_1,38_ = 2.84, *p* = 0.100, *η* ^2^ *p* = 0.07; BF_10_ = 0.974) and the critical Group × Night interaction were not significant (*F*_1,38_ = 1.72, *p* = 0.198, *η* ^2^ *p* = 0.04; BF_10_ = 0.649). The decrease N3% in Night 1 was balanced by an increase in N2% (*F*_1,38_ = 3.66, *p* = 0.063, *η* ^2^ *p* = 0.09; BF_10_ = 1.176), but again the Group (*F*_1,38_ = 4.69, *p* = 0.498, *η* ^2^ *p* = 0.01; BF_10_ = 0.363) and the Group × Night interaction were not significant (*F*_1,38_ = 1.58, *p* = 0.216, *η* ^2^ *p* = 0.04; BF_10_ = 0.680). All the other comparisons were not significant. These results suggest that, although the first night was associated with a lower proportion of time spent in N3 and greater in N2, this effect was similar for the two groups.

## 4 DISCUSSION

The current study aimed to investigate the separate roles of sleep and wakefulness on emotional memory consolidation. Specifically, we aimed to investigate the effect of nocturnal sleep compared with an equal period of daytime wakefulness on subjective emotional reactivity and memory performance for neutral and negative pictures. Moreover, we aimed to assess the effect of delayed sleep‐dependent emotional memory consolidation. Lastly, we explored the effect of nighttime sleep on the encoding of emotional information.

After 12 hr from the encoding, we observed a clear beneficial role of post‐learning sleep compared with post‐learning wakefulness on memory consolidation. However, this beneficial effect of sleep was not specific to negative pictures, suggesting that sleeping after the encoding of visual information reduced the level of forgetting 12 hr later, regardless of stimuli valence. Nevertheless, negative stimuli were better remembered in both conditions, compared with neutral images. Interestingly, 24 hr after the encoding, performance decreased in the SF, who spent the previous 12 hr awake, but not in the WF, who had a night of sleep in the previous 12 hr. We also observed a positive association between memory performance for negative items at the immediate test and REM% the night before the encoding.

Focusing on subjective emotional reactivity, the influence of sleep on valence and arousal levels was not supported by our data. These variables were comparable in WF and SF and did not change across testing sessions, besides a small increase in perceived valence level for the negative pictures from Encoding to T2, likely due to a habituation effect. Furthermore, sleep features recorded the night following the encoding did not seem to be correlated either with memory performance outcomes or with valence and arousal levels.

Regarding the role of sleep on memory consolidation, our data are consistent with existing literature (Diekelmann & Born, 2010; Rasch & Born, 2013), further confirming its positive influence on memory performance. Indeed, we observed an advantage of sleep compared with wakefulness, despite the normal memory decay attributable to the passing of time, as demonstrated by the reduced performance at T2 compared with T1 in both conditions. Moreover, our results resemble the findings of a very recent paper employing the same experimental design, but using a word‐paired association task (Zhang, Whitehurst, & Mednick, 2022). Their main aim was to investigate whether sleep helps to stabilize information by strengthening newly encoded memories against later waking experiences, or to recover these memories after a period of wakefulness. Similar to our study, the authors found better memory retention after 12 hr of sleep compared with a similar period of wakefulness. Moreover, 24 hr after the encoding, both groups (SF and WF) showed the same performance. They also observed a positive association between power in the delta and theta bands in the WF, and change in memory performance from T2 to T3 (i.e. higher power, more information was remembered). They interpret their data suggesting that sleep may help in rescuing degraded memories after a day of wakefulness.

Although our behavioural data are in line with Zhang et al. (2022), we did not find any association between sleep parameters recorded during the night following the encoding and memory performance on the next day. The absence of a clear connection between sleep parameters and memory consolidation is justified by the fact that, despite the recent progress in sleep research, a univocal connection between sleep stages and memory functions has not been found yet (Ackermann & Rasch, 2014). Hence, our results may simply reflect the complexity of sleep processes occurring during the night, rather than the absence of any association. It is also worth considering that in our study we recorded sleep using a portable PSG that did not allow us to assess sleep microstructure (e.g. sleep spindles, slow oscillations, theta power during REM), which may have provided more information about the underlying memory‐related processing during sleep.

The above‐mentioned results refer to memory performance independently from the valence of presented images (i.e. the SF reported a better performance for both neutral and negative images). Indeed, our results did not show any selective, or preferential, effect of sleep on emotional memory performance. This result is in line with what was reported by two recent meta‐analyses on sleep and emotional memories (Lipinska et al., 2019; Schaefer et al., 2020). Both these studies, although they included a different number of studies and used different analytical approaches, indicated that there is no empirical support for the idea that one period of nocturnal or diurnal sleep preferentially consolidates emotional memories. For example, Schaefer et al. (2020) showed an advantage for the consolidation of emotional over neutral material after sleep only in 9 out of 22 studies (40.09%) that compared sleep and wakefulness. These studies suggest that most of the positive effects of sleep on emotional memory occur only under very specific conditions (e.g. using free recall instead of recognition tests, using split‐night and partial sleep deprivation protocols). Schäfer et al. (2020) even suggested, also taking into account studies with no wake condition, that wakefulness, rather than sleep, may be associated with a very small preferential retention of emotional stimuli (i.e. mean standardized mean change ranging from −0.16 to −0.19). Our data are not consistent with this latter idea, but we should stress that our study was not designed to identify an effect size so small.

Nevertheless, we reported a higher memory performance for negative information after 12 and 24 hr, which is coherent with a wide portion of literature that claims that, as negative stimuli are more salient and more activating than neutral ones, their encoding is advantaged (Hu et al., 2006). Indeed, this result is also in line with what was reported by Lipinska et al. (2019), showing that after a period of sleep or wake, participants show a higher performance for negative compared with neutral stimuli, with an effect size similar to what we reported here.

However, the lack of a significant modification of valence and arousal levels across the experimental sessions—and particularly between sleep and wakefulness—partially deviates from the available literature. One of the most well‐known models related to sleep and emotional processing, the Sleep to Forget Sleep to Remember hypothesis (SFSR; Walker & van Der Helm, 2009), proposes that valence and arousal levels of an emotional memory are progressively reduced during REM sleep, whereas the episodic content of that same memory is increasingly consolidated. According to this model, we should have observed a significant reduction of emotional reactivity in SF compared with WF, and also a positive correlation between the magnitude of the reduction and the time spent in REM during the night after the encoding. Despite the validity of the SFSR hypothesis being still widely debated, these results do not necessarily disconfirm it. Firstly, we tested our participants 1 night after the encoding, although several nights of sleep may be required for a significant reduction of valence and arousal levels, even though it has been reported that a single night may be enough in some cases (Van Der Helm et al., 2011). Indeed, the SFSR model explains that emotional reactivity gradually diminishes over time, and only one session of PSG and memory assessment may not be sufficient for complex emotional processing mechanisms to occur. Secondly, it is possible that stimuli were not arousing enough. Therefore, to produce more solid inferences about this model's validity, it would be worthwhile to replicate the present work using more arousing stimuli and testing participants after several nights of sleep.

Regarding the second hypothesis, our results are partially coherent with literature that proposes that sleep could minimize the forgetting of new information even when it occurs long after the learning (Benson & Feinberg, 1977). Indeed, we found that 24 hr after the encoding, the performance of the two groups was comparable. However, some considerations need to be discussed. Firstly, Benson and Feinberg (1977) employed word couples as stimuli, whereas in the present work we employed negative and neutral images. As it is widely established, memory is a complex function divided into several subcategories and, even if words and images are both stored in the declarative memory (Squire, 2004), the specific processes of encoding and consolidation may involve different brain regions and different physiological processes. Secondly, the above‐mentioned authors only considered correct answers as an index for memory performance, whereas the d‐prime index calculated in our study also takes false alarms into account. Indeed, focusing only on the HR, WF had a lower performance compared with SF even 24 hr after the encoding. Thus, the discrepancies in these studies may at least be partially explained. However, our data on d‐prime show that a period of sleep long after the encoding has a positive effect on memory consolidation, in line with the aforementioned‐discussed paper by Zhang et al. (2022). Because WF had a night of rest between T2 and T3, one possible explanation is that sleep performs a protective action on the information that has not been compromised by wakefulness interference. This would support the idea that sleep can rescue damaged and degraded memories from oblivion (Drosopoulos, Schulze, Fischer, & Born, 2007; McDevitt, Duggan, & Mednick, 2015). Conversely, SF underwent an equivalent period of wakefulness between T2 and T3, which may have accelerated the forgetting of information previously “protected” by sleep. However, another interpretation might be considered. Twenty‐four hours after the encoding (T3), both groups might have reached the maximal threshold of memory consolidation, thus preventing any significant modification of memory performance, regardless of the experimental condition.

Finally, the analysis of results obtained the night before encoding in WF seems to support the involvement of REM sleep in emotional memory processing (Goldstein & Walker, 2014; Wagner et al., 2001). The positive relation between memory performance for negative items at T1 and REM sleep percentage the night before the encoding may indicate that REM sleep may optimize the brain for the encoding and the subsequent consolidation of new emotional material, as neutral stimuli seem not to be advantaged by REM. Thus, this result supports the idea that different sleep stages specifically contribute to different memory subcategories. However, because positively valenced images were not employed in this study, it is not possible to infer whether this association is related to emotional memories overall or only to negative ones. This association could also be interesting for clinicians. For example, depressed patients seem to be characterized by both an enhancement of REM sleep (Baglioni et al., 2016) and a negative stimuli bias (Clark & Beck, 2010). Hence, the prolonged increment of REM could exacerbate depressed symptomatology and negative bias or even be a co‐contributing cause. Nevertheless, the negative relation between d‐prime and N2% is more controversial. As widely reported by the literature, N2 has a fundamental role in memory consolidation (Diekelmann & Born, 2010; Rosanova & Ulrich, 2005), but the negative association found in the present study seems to point in the opposite direction, indicating that this stage is disadvantageous for emotional consolidation. However, it is worth considering that the sleep‐monitoring device supplied for this study allowed to precisely measure the duration of each sleep stage, but did not allow us to further investigate sleep micro‐architecture. Moreover, it must be noted that, because sleep duration is fixed, the percentages of the stages vary among subjects. Hence, when a specific stage (e.g. REM) increases its duration, one or more other stages mathematically decrease (e.g. N2). Therefore, our negative correlation may reflect the individual variability of the proportion of sleep stages, thus indicating that participants who report more REM, and automatically less N2, are more inclined to better remember negative information than neutral ones. This latter idea is partially supported by the Bayesian factor, which suggests higher evidence of an association of d‐prime with REM% rather than with N2%. Lastly, we should stress that the interpretation of these correlations is mainly speculative as there was a low number of participants (*n* = 20).

The current results should be interpreted also considering the limitations of this study. Firstly, experimental sessions were entirely performed at participants' homes, thus with limited control of the experimental setting and participants' compliance with the task instructions. However, this same limitation allowed us to conduct a more ecological study. Because participants slept in their beds, they were not subjected to any possible pressure exerted by a laboratory setting, which could have altered the quality of recorded sleep (Agnew Jr, Webb, & Williams, 1966). Nevertheless, they could experience an adaptation night effect. Indeed, when we analysed the sleep pattern in the two groups across the 2 nights, we did observe higher N2% and lower N3% on the first night, but this effect was similar in both groups. Secondly, as mentioned before, recording a single sleep night may not offer sufficient insight into sleep and memory‐related processes, given their huge complexity. Therefore, further studies should record more consecutive sleep nights to overcome this limit. Thirdly, the current study only used static visual stimuli (i.e. pictures) and a recognition task. Therefore, further studies should try to extend our results with different types of stimuli (e.g. video) and different types of tests (e.g. recall). Lastly, we should stress again that the current sample size is quite small (*n* = 20 per group), and our analysis did not have enough power to detect small and more subtle effects. This sample size was chosen based on resource constraints, i.e. we had limited funding, personnel and time to collect the data. Therefore, we defined our sample size using a heuristic procedure (Lakens, 2022), which was based on reviewing the literature about sleep and emotional memory. Considering the 34 studies included in the meta‐analysis by Schäfer and colleagues 2020), the majority of the studies on this topic had a sample size between 12 participants per group or condition (Goldschmied et al., 2015; Wagner, Kashyap, Diekelmann, & Born, 2007) to 24 participants per group or condition (Ashton, Harrington, Smith, & Cairney, 2019), with some exceptions with a larger sample size (Baran et al., 2012; Bennion, Payne, & Kensinger, 2016; Cox et al., 2018). For example, Sopp, Michael, and Mecklinger (2018), using a similar protocol to ours (two groups, three testing periods, a recognition task with neutral and unpleasant stimuli), recruited 23 participants per group, similar to our sample sizes. Therefore, the current results can be used as a comparison with similar studies in the literature, but their interpretation should be limited to effects that are likely medium to large, keeping in mind that our study was not designed to identify small effects (e.g. Cohen's *d* ≤ 0.20). Future studies should replicate and extend our current results with larger sample sizes.

In conclusion, our study confirms the beneficial role of sleep on memory consolidation, either just after the encoding of new information or after a day of wakefulness. Moreover, although negative stimuli have again shown to be better remembered than neutral ones, subjective emotional reactivity seems to not be influenced by a single night of sleep. We also evidenced that memory performance for negative images is positively associated with REM sleep quantity in the night before learning. Overall, sleep seems not to preferentially improve emotional memory, although it may affect the encoding of negative information.

## AUTHOR CONTRIBUTIONS

NC and GC developed the study concept, and contributed to the study design and data collection. All the authors contributed to data analysis, interpreted the data, drafted the manuscript, and approved the final version for submission.

## CONFLICT OF INTEREST

This is not an industry‐supported study. None of the authors has potential conflicts of interest to be disclosed. All authors have seen and approved the manuscript.

## Data Availability

The data that support the findings of this study are available from the corresponding author upon reasonable request.
